# BDNF genotype associated with changes in cortical thickness, severity of symptoms, and cognitive impairments in mild traumatic brain injury

**DOI:** 10.1186/s13041-025-01239-1

**Published:** 2025-10-14

**Authors:** Lei Shi, Yizhen Pan, Jie Yuan, Jue Zhang, Zhiqi Lee, Xuan Li, Haonan Zhang, Xiang Zhang, Tingting Wu, Jierui Ding, Tao Liu, Nengrui Guo, Zhuonan Wang, Lijun Bai

**Affiliations:** 1 The Key Laboratory of Biomedical Information Engineering, Department of Biomedical Engineering, School of Life Science and Technology, Ministry of Education, Xi’an Jiaotong University, Xi’an, 712000 China; 2 Department of Clinical Laboratory, Shuguang Hospital, Shanghai University of Chinese Traditional Medicine, Shanghai, 201203 China; 3 Departments of Radiology, Shuguang Hospital Affiliated to Shanghai University of Traditional Chinese Medicine, Shanghai, 201203 China; 4 Department of Neurosurgery, Shuguang Hospital, Shanghai University of Traditional Chinese Medicine, Shanghai, 201203 China; 5 PET-CT Center, The First Affiliated Hospital of Xi’an Jiaotong University, Xi’an, 710061 China

**Keywords:** Mild traumatic brain injury, Brain-Derived neurotrophic factor, Clinical symptoms, Cognitive impairment, Cortical thickness

## Abstract

**Objective:**

Brain-derived neurotrophic factor (BDNF) is a critical blood protein for brain function; however, its genotypic influence on clinical outcomes and brain structure following mild traumatic brain injury (mTBI) remains unclear. This study investigated the relationship between BDNF polymorphisms and cognitive impairment, symptom severity, and cortical structural injury in mTBI patients.

**Materials and methods:**

Sixty-one mTBI patients underwent neuropsychological assessments and MRI scans within one week post-injury, with 46 patients followed up at one month. Fifty-two healthy controls were included for comparison. Patients with mTBI exhibited clinical symptoms, cognitive impairment, and alterations in cortical thickness during in the acute phase.

**Results:**

BDNF Met gene carriers (*n* = 41) and Val gene carriers (*n* = 20) demonstrated different cognitive performance in the acute phase and exhibited distinct recovery trajectories. Val carriers showed significantly better cognitive flexibility compared to Met carriers (*p* = 0.028) during the acute phase and greater improvement in clinical symptoms at one month (*p* = 0.035). Follow-up MRI scans revealed more extensive and statistically significant alterations in cortical thickness in Met carriers than in Val carriers (*p* < 0.01), particularly in regions associated with cognitive and emotional regulation.

**Conclusion:**

These findings suggest that BDNF polymorphisms in mTBI patients are associated with brain structural changes and may serve as valuable biomarkers for identifying individuals at risk for long-term clinical symptoms and cognitive impairment.

**Supplementary Information:**

The online version contains supplementary material available at 10.1186/s13041-025-01239-1.

## Introduction

Mild traumatic brain injury (mTBI), which accounts for approximately 75% of all traumatic brain injury (TBI) cases, has garnered increasing attention in recent years [1]. This increasing interest is reflected in recent work by Kim et al. [2], which highlights the complex clinical characteristics, neuroimaging findings, and molecular mechanisms associated with military-related mTBI. The prevailing consensus is that mTBI can lead to sustained effects on brain function and behavior. A random-effects meta-analysis revealed that the prevalence of post-concussion symptoms in mTBI patients was 31.3% at 3 to 6 months post-injury [3]. Post-traumatic stress disorder (PTSD) is a psychiatric complication with a relatively high incidence following mTBI and is considered a potential factor contributing to persistent trauma-related issues in mTBI patients [4]. The prevalence of insomnia is significantly higher in individuals who have experienced mTBI compared to estimates reported in the general population [5]. While most individuals with mTBI recover fully within a few months, some patients endure persistent trauma symptoms that impact their daily functioning and professional lives [6]. Research has demonstrated that mTBI disrupts the brain’s microstructure over time, leading to impairments in functional brain networks and potentially triggering severe cognitive deficits, which may underlie the development of clinical symptoms and cognitive impairments [7]. These findings are supported by recent diffusion tensor imaging studies, such as that by Kim et al. [8], which reported white matter alterations in military service members with a history of remote mTBI. Previous studies have shown that the cortical thickness of mTBI patients undergoes significant changes and is correlated with accompanying cognitive impairment [9]. Nevertheless, research on the interaction between clinical manifestations and cognitive outcomes of mTBI, as well as changes in brain structure and function, remains limited. Additionally, the impact of various moderating factors, such as genetic factors, on this interaction is not yet clear [10].

Brain-derived neurotrophic factor (BDNF), located on chromosome 11p13, is a member of the neurotrophin family and plays a crucial role in various brain functions, including synaptic plasticity, neurogenesis, and cell survival [11]. The Val66Met polymorphism in BDNF, which involves a substitution of methionine (Met) for valine (Val) at codon 66, has been linked to diverse neurological outcomes [12]. Research indicates that individuals with the Met al.lele (either BDNF Met/Met or BDNF Met/Val) may experience cognitive impairment, particularly in patients with insomnia [13]. Furthermore, the interaction between BDNF genotypes and mild traumatic brain injury (mTBI) has been shown to influence functional connectivity in key brain regions [14]. Beyond functional connectivity, neuroimaging studies have begun to uncover structural correlates of BDNF genotype variation. For instance, Hayes et al. [15] found that BDNF Met al.lele carriers with a history of mTBI exhibited reduced hippocampal volume, suggesting a genotype-related vulnerability to structural alterations following injury.

Despite these findings, there remains a lack of comprehensive investigation into how BDNF polymorphisms may influence cortical structure—particularly cortical thickness—in mTBI, and how such alterations relate to clinical and cognitive outcomes. This gap in the literature highlights the need to explore neurogenetic markers that may help explain the heterogeneity of post-mTBI recovery trajectories.

In light of these considerations, our study aimed to investigate the relationship between BDNF-Met gene polymorphisms and persistent cognitive impairment, clinical symptoms, and cortical alterations in mTBI. We hypothesized that mTBI patients carrying the Met variant of the BDNF gene may have an increased susceptibility to developing persistent clinical symptoms and cognitive impairments, potentially accompanied by alterations in brain structure. By exploring the utility of BDNF polymorphism as a biomarker for the early identification of high-risk individuals, our research seeks to enhance the understanding of the relationship between genetics, brain injury, and neuropsychological manifestations.

## Materials and methods

### Participants

This prospective study was approved by the Institutional Review Board of Xi’an Jiaotong University (Approval No.2021 − 1381), and written informed consent was obtained from all participants. A total of 61 acute patients (34 males, mean age 33.74 ± 13.51 years, education level 10.21 ± 2.96 years) with mTBI and 52 age-, sex-, and education-matched healthy controls (HC; 24 males, mean age 36.37 ± 11.99 years, education level 11.12 ± 5.95 years) were recruited for this study. At the 1-month follow-up, 46 mTBI patients were reassessed, revealing no significant differences in demographic, clinical, or cluster statistics compared to those who did not return.

The inclusion criteria for patients with mild traumatic brain injury (mTBI) were established based ono the World Health Organization’s Collaborating Centre for Neurotrauma Task Force guidelines [16] and required assessment within one week following the injury. Patients with structural abnormalities identified through conventional neuroimaging, pre-existing conditions (such as headaches, previous brain or spinal injuries, neurological disorders, or substance abuse), or those undergoing psychotropic treatment were excluded from the study. Healthy subjects with no history of acute or chronic pain, neurological or psychiatric disorders, or substance abuse were included in the research.

MRI scans for patients with mild traumatic brain were conducted within 7 days post-injury during the acute phase and at again 1 month post-injury during the sub-acute phase. Clinical and neuropsychological assessments were performed within 24 h of the injury. For healthy MRI and neuropsychological assessments were conducted only once.

### Clinical and neuropsychological assessments

A comprehensive battery of clinical and neuropsychological assessments was administered to all participants, which included: (1) Processing speed, measured by the Trail-Making Test Part A (TMT-A) and the Digit Symbol Coding (DSC) from the Wechsler Adult Intelligence Scale III (WAIS-III) [17]. (2) Working memory and executive function, assessed through the forward and backward Digit Span (FDS and BDS) [18]. (3) Verbal fluency, evaluated using the Verbal (VFT) [19]. (4) Post-concussive symptom severity, measured by the Post-Concussion Syndrome Scale (PSS) [20]. (5) Post-traumatic stress disorder (PTSD) symptom severity, assessed with the PTSD Checklist-Civilian Version (PCL-C) [21]. (6) Insomnia severity, evaluated using the Insomnia Severity Index (ISI) [22].

These assessments were conducted within 24 h of the MRI scan for patients with mTBI in the acute and sub-acute phases. The comprehensive evaluation of cognitive, symptomatic, and functional domains provided a detailed characterization of the clinical profiles of the mTBI patients.

### MRI acquisition and preprocessing

Participants underwent MRI scanning using a GE 750 MRI scanner with the following parameters: TE = 3.43 ms, TR = 7.68 ms, TI = 450 ms, slice thickness = 1 mm, field of view (FOV) = 256 mm × 256 mm, matrix size = 256 × 256, flip angle = 9°, and voxel size = 1.0 mm × 1.0 mm × 1.0 mm, with a total of 188 slices acquired. Subjects were positioned comfortably in the scanner, with their heads restrained to minimize motion, and their heart rate and respiration were monitored throughout the experiment.

### Cortical thickness measurements

Cortical thickness was analyzed using FreeSurfer’s longitudinal pipeline (v7.1.1). For whole-brain exploration, vertex-wise general linear models (GLMs) were performed with cluster-wise family-wise error (FWE) correction (*p* < 0.05). For hypothesis-driven analysis, mean thickness values were extracted from 34 regions per hemisphere (Desikan-Killiany atlas, Table S1) and analyzed using repeated-measures ANOVA, with timepoint (acute/follow-up) as a within-subjects factor, BDNF genotype (Met/Val) as a between-subjects factor, and age, sex, and education as covariates. All statistical tests were FDR-corrected (q < 0.05) for multiple comparisons.

### Genotyping

Based on BDNF genotyping, patients with mTBI were classified into two subgroups: 41 carriers (Met-mTBI group) and 20 carriers of the BDNF^Val/Val^ genotype (Val-mTBI group). Specifically, a 20 mL blood sample was collected from each participant via peripheral venipuncture for DNA genotyping. The samples were centrifuged at 5000 rpm for 10 min, and the plasma was stored at -40 °C until further analysis. Genotyping was performed using a TaqMan SNP genotyping assay to identify the BDNF^Met/Met^, BDNF^Met/Val^, and BDNF^Val/Val^ genotypes.

### Statistical analysis

Statistical analysis was conducted using SPSS version 25.0. The normality of the data was assessed using the Shapiro–Wilk W-test. For normally distributed data, between-group comparisons were performed using a two-sample t-test, while non-normally distributed data were analyzed using the Mann–Whitney U test. One-way ANOVA, followed by Bonferroni post hoc tests, was employed to evaluate statistical significance across more than two groups. Specifically, one-way ANOVA was used to investigate potential differences in clinical symptom severity and cognitive performance among mTBI patients with various BDNF gene subtypes. Categorical data were analyzed using the chi-square test. Sex, age, and years of education were treated as covariates and regressed using linear regression. A p-value of less than 0.05 was considered statistically significant, and false discovery rate (FDR) correction with q < 0.05 was applied for multiple comparisons in all analyses.

## Results

### Demographic, clinical symptoms and cognitive characteristics

There were no significant differences in the demographic characteristics of mTBI and HC groups (sex: *p* = 0.310; age: *p* = 0.280; years of education: *p* = 0.323). However, significant differences were observed between the mTBI and HC groups on various cognitive and clinical measures, including DSC (*p* = 0.027), PSS (*p* < 0.001), PCL-C (*p* < 0.001), and ISI (*p* < 0.001). These results indicate that patients with mTBI exhibited slower information processing speed, mild PTSD symptoms, sequelae of traumatic brain injury, and increased insomnia symptoms within 7 days post-injury (Table 1).

**Table 1:** Demographic and cognitive characteristics for mTBI and HC participants

Items	mTBI Group (*n* = 61)	HC Group (*n* = 52)	*p* value
Male, n (%)	34 (55.7)	24 (46.2)	0.310
Age, y	33.74 ± 13.51	36.37 ± 11.99	0.280
Education, y	10.21 ± 2.96	11.12 ± 5.95	0.323
TMT-A	52.25 ± 30.38	47.17 ± 33.65	0.402
DSC	38.93 ± 13.92	45.73 ± 17.64	0.027
FDS	8.20 ± 1.45	8.13 ± 1.53	0.825
BDS	4.26 ± 1.48	4.33 ± 1.77	0.833
VFT	17.11 ± 5.47	18.40 ± 6.05	0.237
PSS	9.44 ± 6.82	2.58 ± 2.97	< 0.001
PCL-C	24.25 ± 6.02	17.00 ± 0.00	< 0.001
ISI	6.69 ± 5.97	2.04 ± 3.03	< 0.001

TMT-A, Trail making A; DSC, Digit Symbol Coding Score; FDS, Forward Digit Span; BDS, Backward Digit Span; VFT, Verbal Fluency Test; PSS, Post-Concussion Syndrome Scale; PCL-C, PCL-C, Post-traumatic stress disorder (PTSD) symptom severity: PTSD Checklist-Civilian Version; ISI, Insomnia Severity Index

### Cognitive impairment in mTBI patients carrying the Met gene

BDNF genotyping revealed that there were 41 carriers of the BDNF Met/Met and BDNF Met/Val genotypes (Met subgroup), and 20 carriers of the BDNF Val/Val genotype (Val-mTBI group) within the mTBI group. The healthy control (HC) group comprised 36 carriers of the BDNF Met/Met, and BDNF Met/Val genotypes, along with 16 carriers of the BDNF Val/Val genotype. The Hardy-Weinberg Equilibrium test indicated no evidence of population stratification or genotyping errors.

**Fig. 1:**
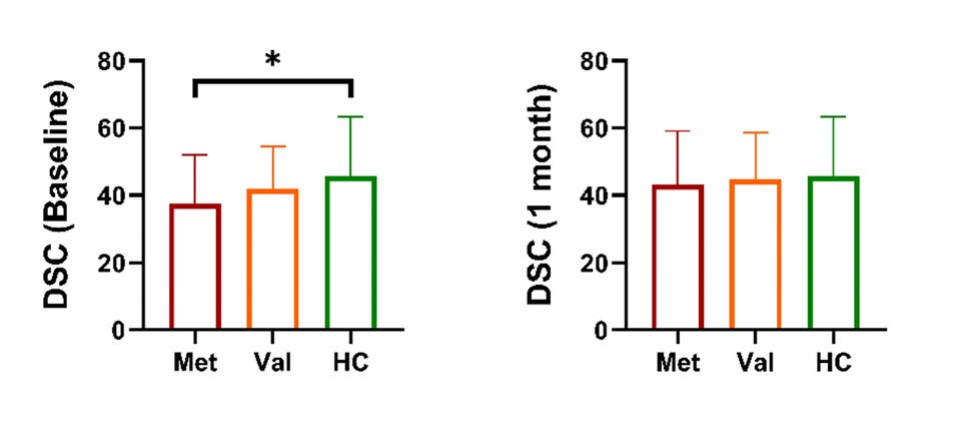
Between-group comparison of Digit Symbol Coding (DSC) test in HCs and patients carrying different BDNF genes in the acute phase and the 1-month follow-up phase. The ∗ indicates the significant difference with *p* value between 0.01 and 0.05. Met, Met-mTBI patients; Val, Val-mTBI patients

The DSC score in the Met-mTBI group was significantly lower than that of the healthy control (HC) group (*p* = 0.043) during the acute phase following the injury, although the abnormal DSC scores improved during the follow-up period (see Fig. 1). In contrast, the Val-mTBI group did not exhibit abnormal DSC scores during either the acute or sub-acute phases. The BDNF^Val/Val^ group showed higher DSC scores (45.2 ± 6.8) compared to the Met carrier group (38.7 ± 5.4; *p* = 0.043).

### Persistent clinical symptoms in mTBI patients carrying Met gene

**Fig. 2:**
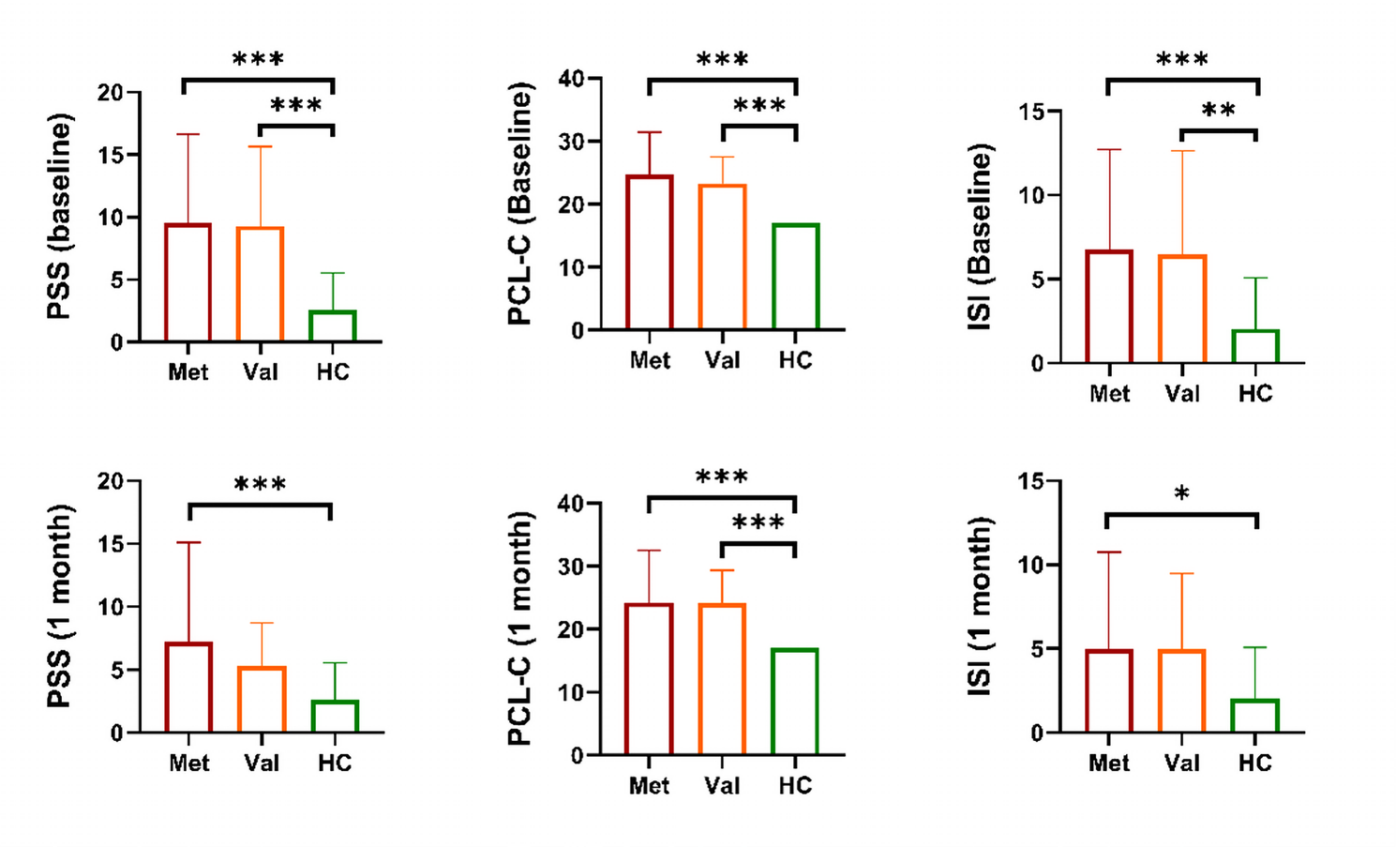
Between-group comparison of neuropsychological symptoms in HCs and patients carrying different BDNF genes in the acute phase and the 1-month follow-up phase. The ∗ indicates the significant difference with *p* value between 0.01 and 0.05, the ∗∗ displays the *p* value between 0.001 and 0.01, and the ∗∗∗ represents the *p* ≤ 0.001. Met, Met-mTBI patients; Val, Val-mTBI patients; PSS, Post-Concussion Syndrome Scale; PCL-C, Post-traumatic stress disorder (PTSD) symptom severity: PTSD Checklist-Civilian Version; ISI, Insomnia Severity Index

One-way ANOVA was employed to investigate potential differences in clinical symptom severity among mTBI patients with various gene subtypes. PCL-C scores were significantly higher in patients at each stage for both genotypes compared to the healthy control (HC) group (*p* < 0.001). Additionally, the initial PSS and ISI scores for both genotypes were significantly elevated in mTBI patients compared to the HC group (PSS for both subgroups: *p* < 0.001; ISI for the Met -mTBI group: *p* < 0.001; ISI of Val-mTBI group: *p* = 0.001). However, during the subacute phase (1-month follow-up), only Met-mTBI patients continued to show significantly increased PSS and ISI scores compared to the HC group (PSS: *p* < 0.001; ISI: *p* = 0.010). The PSS and ISI scores for the Val-mTBI group returned to normal levels (PSS: *p* = 0.255; ISI: *p* = 0.079) (Fig. 2). At 1-month follow-up, Val/Val carriers’ PSS scores decreased to HC levels (*p* = 0.255), while Met carriers maintained elevated scores (*p* < 0.001).

### Relationship between BDNF polymorphisms and cortical thickness following mTBI

**Fig. 3:**
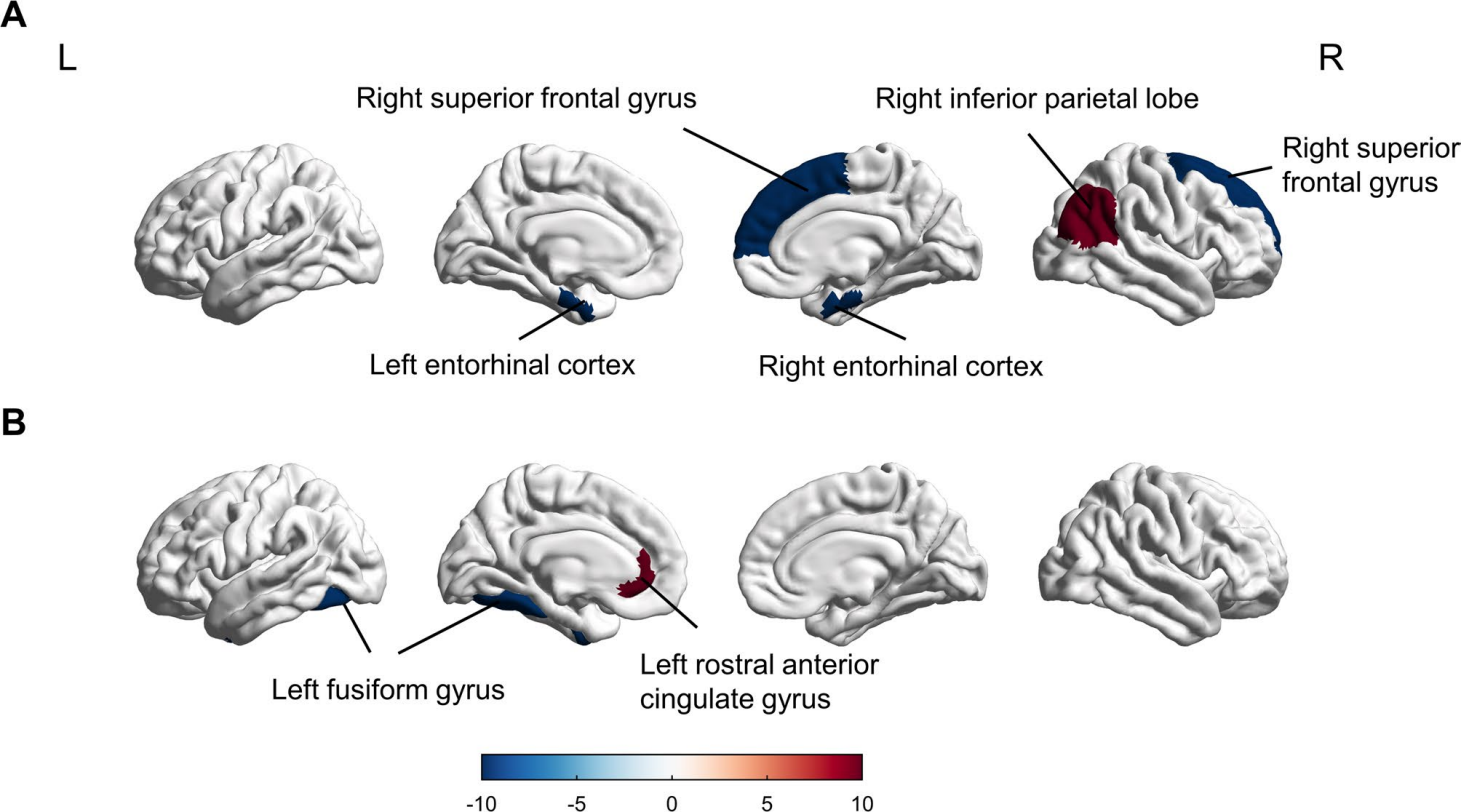
Group differences in cortical thickness between mTBI patients and healthy controls (HC) in the acute phase (**A**) and at 1-month post-injury (**B**). Red-yellow indicates cortical thinning in mTBI (values represent thickness reduction in mm); blue-cyan indicates increased thickness where present. Results are thresholded at *p* < 0.05 (FDR-corrected). Significant regions are labeled according to the Desikan-Killiany atlas (e.g., frontal cortex, precuneus)

Compared to healthy controls (HC), patients with acute mTBI exhibited significantly reduced cortical thickness in the bilateral entorhinal cortex (*p* = 0.032) and the right superior frontal gyrus (*p* = 0.027). Conversely, cortical thickness in the right inferior parietal gyrus (Brodmann area 40; MNI coordinates: x = 50, y=-30, z = 30) increased by 0.3 mm (± 0.1) compared to HC (*p* = 0.041) (Fig. 3A, Table S2). Longitudinal analysis showed no significant thickness differences in the right inferior parietal gyrus between mTBI and HC groups at follow-up (*p* = 0.049). However, a significant reduction in cortical thickness was observed in the left fusiform gyrus (*p* = 0.022), while an increase in cortical thickness was found in the left rostral anterior cingulate gyrus (*p* = 0.038) (Fig. 3B, Table S2).

**Fig. 4:**
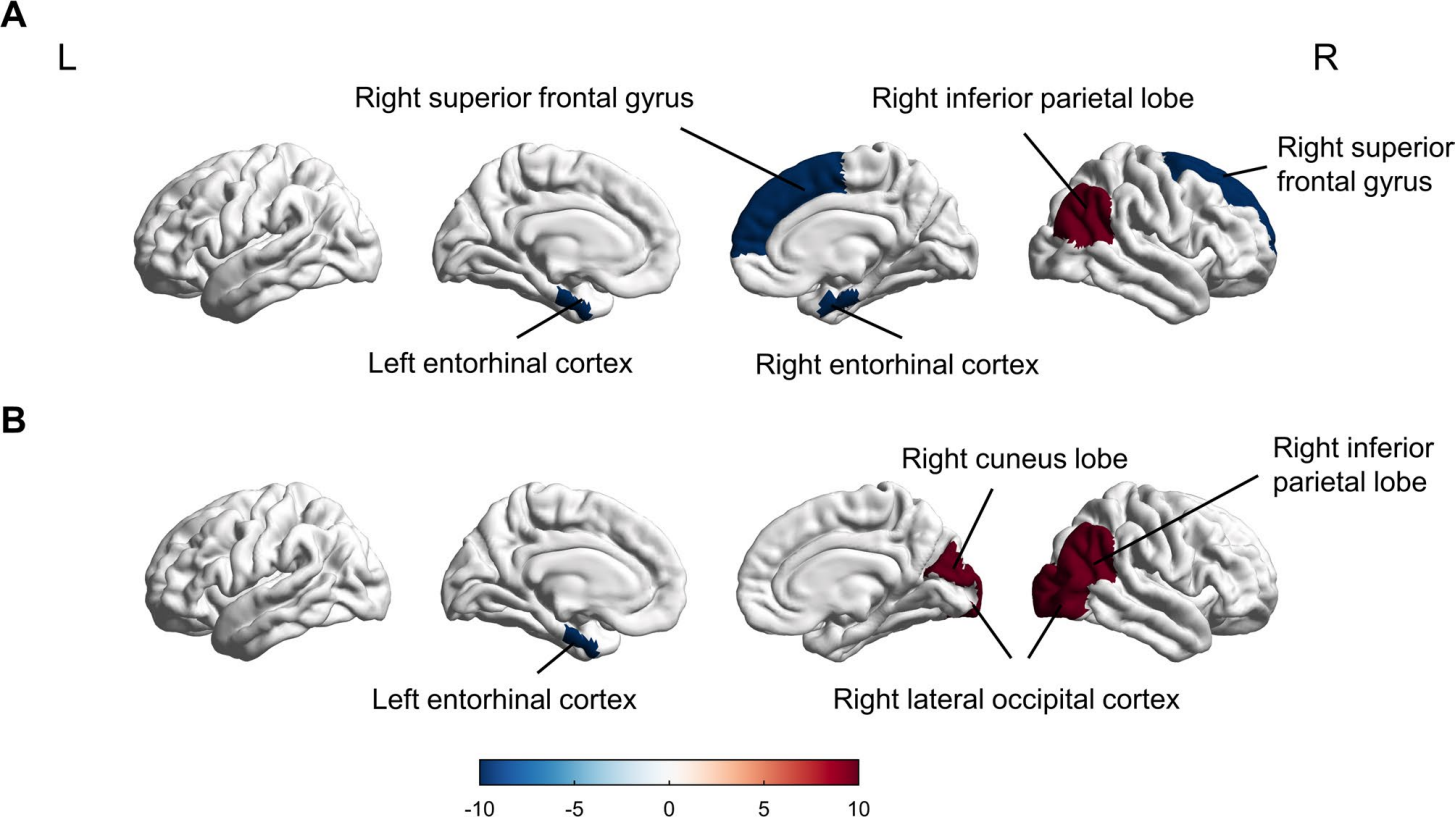
Cortical thickness differences between Met-mTBI (**A**) and Val-mTBI (**B**) patients compared to healthy controls (HC) in the acute phase. Warm colors (red-yellow) indicate cortical thinning in patient subgroups while cool colors (blue-cyan) represent cortical thickening where present, with the scale bar displaying thickness differences in millimeters. Statistical significance was set at *p* < 0.05 (FDR-corrected). Anatomically significant clusters are explicitly labeled, including characteristic findings such as “Met-mTBI: Thinning in left temporal lobe” and “Val-mTBI: Thinning in right frontal cortex”, with all regional identifications based on standard neuroanatomical atlases

**Fig. 5:**
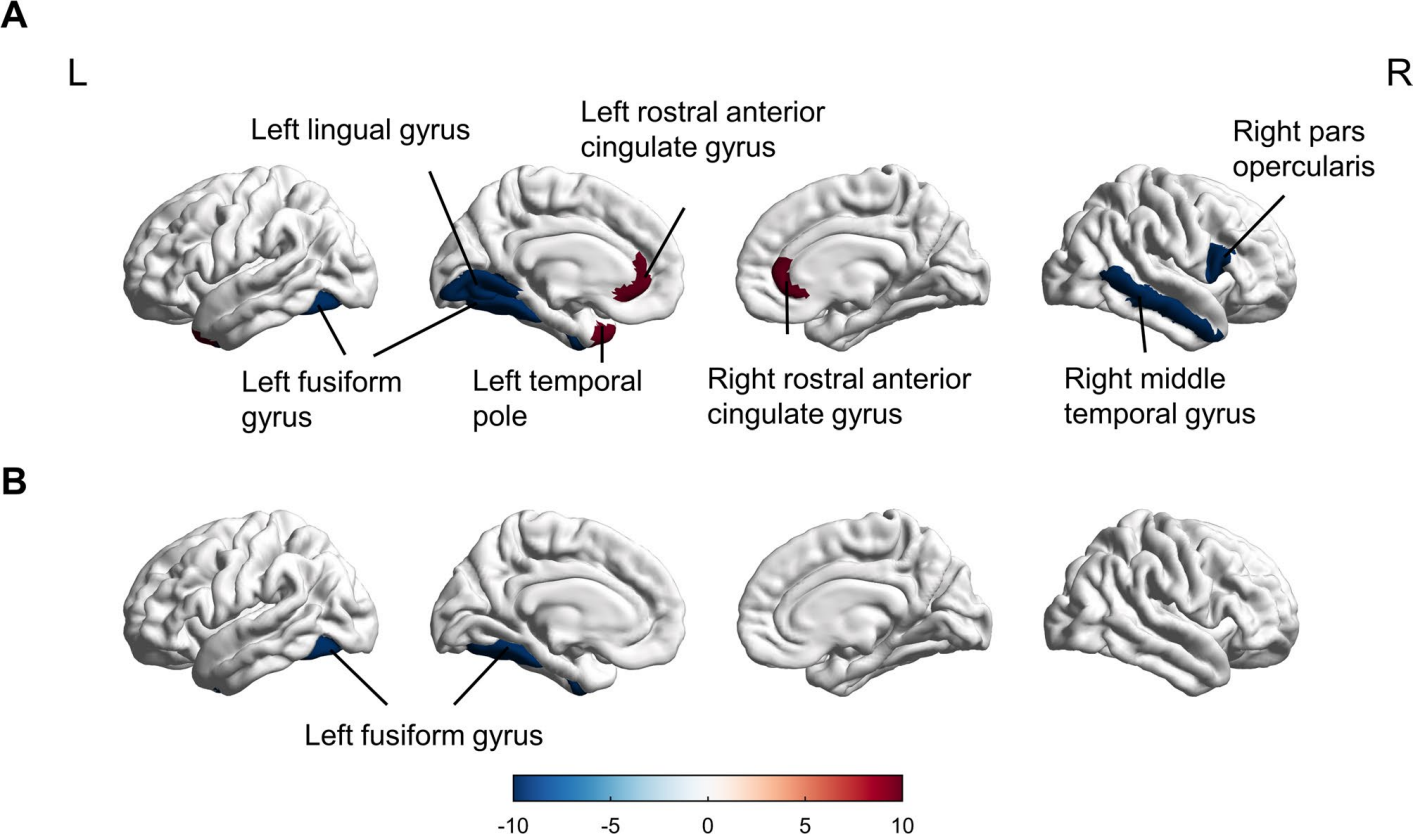
Cortical thickness differences between Met-mTBI (**A**) and Val-mTBI (**B**) patients compared to healthy controls (HC) at 1-month post-injury, using the same color scale as Fig. 4 (red-yellow for thinning, blue-cyan for thickening) with an explicit scale range of -0.2 to + 0.2 mm. Statistical thresholds were maintained consistent with Fig. 3 (*p* < 0.05, FDR-corrected). Key regions showing longitudinal changes are prominently labeled, including “Met-mTBI: Persistent thinning in parietal cortex” and “Val-mTBI: Recovery in frontal regions”, with anatomical identification based on the Desikan-Killiany atlas

When examining the effects of BDNF genotype, consistent patterns were observed in patients with mTBI carrying the Met allele (*p* = 0.005 for the left fusiform gyrus). In contrast, the Val-mTBI group exhibited decreased cortical thickness solely in the left entorhinal cortex (*p* = 0.045), while increased cortical thickness was noted in the right cuneus, right lateral occipital cortex, and right inferior parietal lobe (*p* = 0.032, *p* = 0.041, *p* = 0.027, respectively) (Fig. 4, Table S3). During the follow-up stage, mTBI patients with the Met allele demonstrated widespread alterations in cortical thickness across multiple regions. Representative examples include: (1) decreased thickness in the left fusiform gyrus (2.45 ± 0.12 mm vs. HC 2.68 ± 0.15 mm, *p* = 0.008) and right pars opercularis (2.31 ± 0.11 mm vs. HC 2.52 ± 0.13 mm, *p* = 0.012); (2) increased thickness in the bilateral rostral anterior cingulate gyrus (left: 3.12 ± 0.14 mm vs. HC 2.89 ± 0.16 mm, *p* = 0.009; right: 3.08 ± 0.15 mm vs. HC 2.85 ± 0.17 mm, *p* = 0.011) (Fig. 5A, Table S3). In contrast, Val-mTBI patients showed only decreased cortical thickness in the left fusiform gyrus compared to healthy controls (HC) one month post-injury (Fig. 5B, Table S3). Val/Val carriers showed cortical thickness changes in 1 region (left fusiform gyrus), whereas Met carriers exhibited changes in 5 regions (Fig. 5).

## Discussion

This study investigated the relationships among BDNF genotype, cortical thickness changes, clinical symptoms, and cognitive dysfunction in patients with mild traumatic brain injury (mTBI). Our main findings are as follows: (1) Compared to healthy controls, mTBI patients exhibited cognitive impairments, post-concussion symptoms, post-traumatic stress disorder (PTSD), and insomnia in the acute phase, with these impairments showing genotype-dependent recovery patterns. (2) Patients carrying the BDNF Met allele demonstrated poorer recovery of cognitive flexibility and clinical symptoms than Val homozygotes. (3) Met carriers exhibited more extensive and sustained cortical thickness alterations, whereas Val carriers showed more localized changes, such as reduced thickness in the left fusiform gyrus at one month post-injury.

Consistent with prior studies [23–25], we confirmed that mTBI induces acute neurobehavioral sequelae including cognitive dysfunction, PTSD symptoms, and sleep disturbances. Such impairments can severely disrupt daily life and reduce quality of life. Previous research in war veterans with PTSD demonstrated significantly lower plasma BDNF levels and worse cognitive outcomes compared to healthy controls [26], suggesting plasma BDNF as a potential biomarker of cognitive decline. Similarly, in our cohort, only Met al.lele carriers exhibited persistent symptomatology and decreased cognitive flexibility at follow-up, highlighting the genetic contribution of BDNF polymorphisms to recovery trajectories.

Our findings that the Val/Val genotype is associated with better cognitive outcomes align with prior evidence linking the Val allele to enhanced neuroplasticity and cognitive resilience after brain injury [27, 28]. The Val allele may promote more effective neural repair and synaptic remodeling, whereas the Met al.lele impairs activity-dependent BDNF secretion, thereby diminishing neuroplasticity and prolonging dysfunction [29]. These genotype effects likely underlie individual variability in clinical and cognitive recovery post-mTBI.

Importantly, our neuroimaging results provide mechanistic insights by revealing genotype-dependent cortical thickness alterations linked to specific brain-behavior relationships. The anterior cingulate cortex (ACC) showed increased thickness at follow-up in Met carriers. Given the ACC’s central role in emotional regulation, cognitive control, and error monitoring, and its known involvement in PTSD and affective symptoms [30], the observed thickening may reflect maladaptive neuroplasticity or compensatory responses contributing to persistent emotional dysregulation in Met-mTBI patients.

In contrast, the fusiform gyrus exhibited reduced thickness primarily in Val carriers at one month. Traditionally associated with visual object and face recognition, emerging evidence implicates the fusiform in working memory and executive functions frequently impaired in mTBI [31]. Thus, cortical thinning here may underlie deficits in higher-order cognitive processes. Additionally, the entorhinal cortex, affected during the acute phase, is a key hub for episodic memory formation and emotional encoding [32], suggesting its early involvement contributes to memory complaints and affective disturbances after injury.

BDNF plays a crucial role in maintaining synaptic plasticity and structural integrity in these regions, especially within frontal-limbic circuits encompassing the ACC and temporal lobe. The Met al.lele’s reduction of activity-dependent BDNF secretion impairs neuroplasticity and disrupts connectivity, potentially explaining the more extensive and persistent cortical alterations and clinical symptoms in Met carriers [33, 34]. Conversely, Val carriers may benefit from preserved BDNF signaling that supports neural repair and recovery.

The distinct recovery patterns observed between Met and Val carriers can thus be attributed to their differential effects on neuroplasticity and neuroprotection. The Met al.lele’s genetic vulnerability predisposes individuals to sustained synaptic dysfunction and neuronal loss after mTBI, resulting in poorer clinical outcomes [35, 36]. The Val allele enhances resilience and recovery capacity, consistent with its association with superior cognitive performance and reduced susceptibility to neurological disorders [37, 38].

This study has several limitations. First, we focused on acute and subacute stages post-injury; long-term trajectories of cortical changes and their clinical correlates require further investigation. Second, our relatively small sample size, especially in the Val-mTBI group, may limit generalizability. Third, we did not perform direct correlations between regional cortical thickness and quantitative cognitive or clinical measures, an important direction for future studies. Finally, the complex interplay of genetic, structural, and clinical factors necessitates multimodal approaches to better understand and manage mTBI.

## Conclusion

In summary, our results demonstrate that BDNF genotype significantly influences cortical thickness alterations, symptom severity, and cognitive recovery in mTBI. Met allele carriers show more widespread cortical structural changes and poorer clinical outcomes than Val homozygotes. These findings highlight BDNF genotyping as a promising biomarker to identify individuals at risk for prolonged post-mTBI deficits and to guide personalized therapeutic strategies aimed at enhancing neuroplasticity and recovery.

## Supplementary Information

Below is the link to the electronic supplementary material.

Supplementary Material 1

## Data Availability

No datasets were generated or analysed during the current study.

## References

[1] Bergman K, Given B, Fabiano R, Schutte D, von Eye A, Davidson S. Symptoms associated with mild traumatic brain injury/concussion: the role of bother. J Neurosci Nurs. 2013;45(3):124–32. 23558978 10.1097/JNN.0b013e31828a418b

[2] Kim SY, Yeh PH, Ollinger JM, Morris HD, Hood MN, Ho VB, Choi KH. Military-related mild traumatic brain injury: clinical characteristics, advanced neuroimaging, and molecular mechanisms. Transl Psychiatry. 2023;13(1):289. 37652994 10.1038/s41398-023-02569-1 PMC10471788

[3] Cancelliere C, Verville L, Stubbs JL, Yu H, Hincapié CA, Cassidy JD, Wong JJ, Shearer HM, Connell G, Southerst D, et al. Post-Concussion symptoms and disability in adults with mild traumatic brain injury: A systematic review and Meta-Analysis. J Neurotrauma. 2023;40(11–12):1045–59. 36472218 10.1089/neu.2022.0185

[4] O’Neil ME, Klyce DW, Pogoda TK, Cifu DX, Eggleston BE, Cameron DC, Wilde EA, Walker WC, Carlson KF. Associations among PTSD and postconcussive symptoms in the Long-Term impact of Military-Relevant brain injury consortium-Chronic effects of neurotrauma consortium prospective, longitudinal study cohort. J Head Trauma Rehabil. 2021;36(6):E363–72. 33656490 10.1097/HTR.0000000000000665

[5] Montgomery MC, Baylan S, Gardani M. Prevalence of insomnia and insomnia symptoms following mild-traumatic brain injury: A systematic review and meta-analysis. Sleep Med Rev. 2022;61:101563. 35033968 10.1016/j.smrv.2021.101563

[6] Marshall S, Bayley M, McCullagh S, Velikonja D, Berrigan L, Ouchterlony D, Weegar K. Updated clinical practice guidelines for concussion/mild traumatic brain injury and persistent symptoms. Brain Inj. 2015;29(6):688–700. 25871303 10.3109/02699052.2015.1004755

[7] De Simoni S, Jenkins PO, Bourke NJ, Fleminger JJ, Hellyer PJ, Jolly AE, Patel MC, Cole JH, Leech R, Sharp DJ. Altered caudate connectivity is associated with executive dysfunction after traumatic brain injury. Brain. 2018;141(1):148–64. 29186356 10.1093/brain/awx309 PMC5837394

[8] Kim S, Ollinger J, Song C, Raiciulescu S, Seenivasan S, Wolfgang A, Werner JK, Yeh PH. White matter alterations in military service members with remote mild traumatic brain injury. JAMA Netw Open. 2024;7(4):e248121. 38635266 10.1001/jamanetworkopen.2024.8121 PMC11161843

[9] Santhanam P, Wilson SH, Oakes TR, Weaver LK. Accelerated age-related cortical thinning in mild traumatic brain injury. Brain Behav. 2019;9(1):e01161. 30488646 10.1002/brb3.1161 PMC6346670

[10] Bennett ER, Reuter-Rice K, Laskowitz DT. Frontiers in Neuroscience Genetic Influences in Traumatic Brain Injury. In: *Translational Research in Traumatic Brain Injury.* Edited by Laskowitz D, Grant G. Boca Raton (FL): CRC Press/Taylor and Francis Group© 2016 by Taylor & Francis Group, LLC.; 2016. 26583176

[11] Wang CS, Kavalali ET, Monteggia LM. BDNF signaling in context: from synaptic regulation to psychiatric disorders. Cell. 2022;185(1):62–76. 34963057 10.1016/j.cell.2021.12.003 PMC8741740

[12] Jayasekeran V, Pendleton N, Holland G, Payton A, Jefferson S, Michou E, Vasant D, Ollier B, Horan M, Rothwell J et al. Val66Met in brain-derived neurotrophic factor affects stimulus-induced plasticity in the human pharyngeal motor cortex. *Gastroenterology* 2011, 141(3):827–836.e821-823. 10.1053/j.gastro.2011.05.047 21699787

[13] Zaki NFW, Saleh E, Elwasify M, Mahmoud E, Zaki J, Spence DW, BaHammam AS, Pandi-Perumal SR. The association of BDNF gene polymorphism with cognitive impairment in insomnia patients. Prog Neuropsychopharmacol Biol Psychiatry. 2019;88:253–64. 30076879 10.1016/j.pnpbp.2018.07.025

[14] Fleischer AW, Fox LC, Davies DR, Vinzant NJ, Scholl JL, Forster GL. Sub-region expression of brain-derived neurotrophic factor in the dorsal hippocampus and amygdala is affected by mild traumatic brain injury and stress in male rats. Heliyon. 2024;10(1):e23339. 38169784 10.1016/j.heliyon.2023.e23339 PMC10758828

[15] Hayes JP, Reagan A, Logue MW, Hayes SM, Sadeh N, Miller DR, Verfaellie M, Wolf EJ, McGlinchey RE, Milberg WP, et al. BDNF genotype is associated with hippocampal volume in mild traumatic brain injury. Genes Brain Behav. 2018;17(2):107–17. 28755387 10.1111/gbb.12403 PMC5787402

[16] Holm L, Cassidy JD, Carroll LJ, Borg J. Summary of the WHO collaborating centre for neurotrauma task force on mild traumatic brain injury. J Rehabil Med. 2005;37(3):137–41. 16040469 10.1080/16501970510027321

[17] González-Blanch C, Pérez-Iglesias R, Rodríguez-Sánchez JM, Pardo-García G, Martínez-García O, Vázquez-Barquero JL, Crespo-Facorro B. A digit symbol coding task as a screening instrument for cognitive impairment in first-episode psychosis. Arch Clin Neuropsychol. 2011;26(1):48–58. 21134887 10.1093/arclin/acq086

[18] Jacola LM, Willard VW, Ashford JM, Ogg RJ, Scoggins MA, Jones MM, Wu S, Conklin HM. Clinical utility of the N-back task in functional neuroimaging studies of working memory. J Clin Exp Neuropsychol. 2014;36(8):875–86. 25252868 10.1080/13803395.2014.953039 PMC4229404

[19] Matsuo K, Taneichi K, Matsumoto A, Ohtani T, Yamasue H, Sakano Y, Sasaki T, Sadamatsu M, Kasai K, Iwanami A, et al. Hypoactivation of the prefrontal cortex during verbal fluency test in PTSD: a near-infrared spectroscopy study. Psychiatry Res. 2003;124(1):1–10. 14511791 10.1016/s0925-4927(03)00093-3

[20] Merz ZC, Zane K, Emmert NA, Lace J, Grant A. Examining the relationship between neuroticism and post-concussion syndrome in mild traumatic brain injury. Brain Inj. 2019;33(8):1003–11. 30810394 10.1080/02699052.2019.1581949

[21] Karstoft KI, Andersen SB, Bertelsen M, Madsen T. Diagnostic accuracy of the posttraumatic stress disorder checklist-civilian version in a representative military sample. Psychol Assess. 2014;26(1):321–5. 24188155 10.1037/a0034889

[22] Seo JG, Park SP. Significance of fatigue in patients with migraine. J Clin Neurosci. 2018;50:69–73. 29396068 10.1016/j.jocn.2018.01.032

[23] Pavlovic D, Pekic S, Stojanovic M, Popovic V. Traumatic brain injury: neuropathological, neurocognitive and neurobehavioral sequelae. Pituitary. 2019;22(3):270–82. 30929221 10.1007/s11102-019-00957-9

[24] Ashina H, Al-Khazali HM, Iljazi A, Ashina S, Amin FM, Lipton RB, Schytz HW. Psychiatric and cognitive comorbidities of persistent post-traumatic headache attributed to mild traumatic brain injury. J Headache Pain. 2021;22(1):83. 34311696 10.1186/s10194-021-01287-7 PMC8314480

[25] Kim SY, Soumoff AA, Raiciulescu S, Kemezis PA, Spinks EA, Brody DL, Capaldi VF, Ursano RJ, Benedek DM, Choi KH. Association of traumatic brain injury severity and Self-Reported neuropsychiatric symptoms in wounded military service members. Neurotrauma Rep. 2023;4(1):14–24. 36726873 10.1089/neur.2022.0063 PMC9886188

[26] Domitrovic Spudic S, Nikolac Perkovic M, Uzun S, Nedic Erjavec G, Kozumplik O, Svob Strac D, Mimica N, Pivac N. Reduced plasma BDNF concentration and cognitive decline in veterans with PTSD. Psychiatry Res. 2022;316:114772. 35961151 10.1016/j.psychres.2022.114772

[27] Krueger F, Pardini M, Huey ED, Raymont V, Solomon J, Lipsky RH, Hodgkinson CA, Goldman D, Grafman J. The role of the Met66 brain-derived neurotrophic factor allele in the recovery of executive functioning after combat-related traumatic brain injury. J Neurosci. 2011;31(2):598–606. 21228168 10.1523/JNEUROSCI.1399-10.2011 PMC3195417

[28] Merritt VC, Clark AL, Evangelista ND, Sorg SF, Schiehser DM, Delano-Wood L. Dissociation of BDNF Val66Met polymorphism on neurocognitive functioning in military veterans with and without a history of remote mild traumatic brain injury. Clin Neuropsychol. 2020;34(6):1226–47. 32204647 10.1080/13854046.2020.1740324 PMC7415574

[29] Treble-Barna A, Wade SL, Pilipenko V, Martin LJ, Yeates KO, Taylor HG, Kurowski BG. Brain-Derived neurotrophic factor Val66Met and behavioral adjustment after early childhood traumatic brain injury. J Neurotrauma. 2022;39(1–2):114–21. 33605167 10.1089/neu.2020.7466 PMC8785712

[30] Sheth C, Rogowska J, Legarreta M, McGlade E, Yurgelun-Todd D. Functional connectivity of the anterior cingulate cortex in veterans with mild traumatic brain injury. Behav Brain Res. 2021;396:112882. 32853657 10.1016/j.bbr.2020.112882

[31] Chen CJ, Wu CH, Liao YP, Hsu HL, Tseng YC, Liu HL, Chiu WT. Working memory in patients with mild traumatic brain injury: functional MR imaging analysis. Radiology. 2012;264(3):844–51. 22829681 10.1148/radiol.12112154

[32] Chen Y, Monaco S, Byrne P, Yan X, Henriques DY, Crawford JD. Allocentric versus egocentric representation of remembered reach targets in human cortex. J Neurosci. 2014;34(37):12515–26. 25209289 10.1523/JNEUROSCI.1445-14.2014 PMC6615499

[33] Oane I, Barborica A, Mindruta IR. Cingulate cortex: anatomy, structural and functional connectivity. J Clin Neurophysiol. 2023;40(6):482–90. 36930223 10.1097/WNP.0000000000000970

[34] Kunimatsu A, Yasaka K, Akai H, Kunimatsu N, Abe O. MRI findings in posttraumatic stress disorder. J Magn Reson Imaging. 2020;52(2):380–96. 31515885 10.1002/jmri.26929

[35] Mercado NM, Stancati JA, Sortwell CE, Mueller RL, Boezwinkle SA, Duffy MF, Fischer DL, Sandoval IM, Manfredsson FP, Collier TJ, et al. The BDNF Val66Met polymorphism (rs6265) enhances dopamine neuron graft efficacy and side-effect liability in rs6265 knock-in rats. Neurobiol Dis. 2021;148:105175. 33188920 10.1016/j.nbd.2020.105175 PMC7855552

[36] Xu X, Garcia J, Ewalt R, Nason S, Pozzo-Miller L. The BDNF val-66-met polymorphism affects neuronal morphology and synaptic transmission in cultured hippocampal neurons from Rett syndrome mice. Front Cell Neurosci. 2017;11:203. 28751857 10.3389/fncel.2017.00203 PMC5508027

[37] Redlich R, Schneider I, Kerkenberg N, Opel N, Bauhaus J, Enneking V, Repple J, Leehr EJ, Grotegerd D, Kähler C, et al. The role of BDNF methylation and Val(66) Met in amygdala reactivity during emotion processing. Hum Brain Mapp. 2020;41(3):594–604. 31617281 10.1002/hbm.24825 PMC7268057

[38] Harrisberger F, Smieskova R, Schmidt A, Lenz C, Walter A, Wittfeld K, Grabe HJ, Lang UE, Fusar-Poli P, Borgwardt S. BDNF Val66Met polymorphism and hippocampal volume in neuropsychiatric disorders: A systematic review and meta-analysis. Neurosci Biobehav Rev. 2015;55:107–18. 25956254 10.1016/j.neubiorev.2015.04.017

