# Supplementary material for: BDNF genotype associated with changes in cortical thickness, severity of symptoms, and cognitive impairments in mild traumatic brain injury
Source: Mol Brain. 2025 Oct 14;18:79. doi: 10.1186/s13041-025-01239-1 (PMC12522302; doi:10.1186/s13041-025-01239-1)
Supplement: Supplementary file 1 — Supplementary Material 1 [file 13041_2025_1239_MOESM1_ESM.docx]

**Table S1.** Brain template partitioning based on Desikan-Killiany

| Labels | Abbreviation of partitions | Full name of the partitions |
| --- | --- | --- |
| 1/35 | bankssts | Banks superior temporal sulcus |
| 2/36 | caudalanteriorcingulate | Caudal anterior-cingulate cortex |
| 3/37 | caudalmiddlefrontal | Caudal middle frontal gyrus |
| 4/38 | cuneus | Cuneus cortex |
| 5/39 | entorhinal | Entorhinal cortex |
| 6/40 | fusiform | Fusiform gyrus |
| 7/41 | inferiorparietal | Inferior parietal cortex |
| 8/42 | inferiortemporal | Inferior temporal gyrus |
| 9/43 | isthmuscingulate | Isthmus–cingulate cortex |
| 10/44 | lateraloccipital | Lateral occipital cortex |
| 11/45 | lateralorbitofrontal | Lateral orbital frontal cortex |
| 12/46 | lingual | Lingual gyrus |
| 13/47 | medialorbitofrontal | Medial orbital frontal cortex |
| 14/48 | middletemporal | Middle temporal gyrus |
| 15/49 | parahippocampal | Parahippocampal gyrus |
| 16/50 | paracentral | Paracentral lobule |
| 17/51 | parsopercularis | Pars opercularis |
| 18/52 | parsorbitalis | Pars orbitalis |
| 19/53 | parstriangularis | Pars triangularis |
| 20/54 | pericalcarine | Pericalcarine cortex |
| 21/55 | postcentral | Postcentral gyrus |
| 22/56 | posteriorcingulate | Posterior-cingulate cortex |
| 23/57 | precentral | Precentral gyrus |
| 24/58 | precuneus | Precuneus cortex |
| 25/59 | rostralanteriorcingulate | Rostral anterior cingulate cortex |
| 26/60 | rostralmiddlefrontal | Rostral middle frontal gyrus |
| 27/61 | superiorfrontal | Superior frontal gyrus |
| 28/62 | superiorparietal | Superior parietal cortex |
| 29/63 | superiortemporal | Superior temporal gyrus |
| 30/64 | supramarginal | Supramarginal gyrus |
| 31/65 | frontalpole | Frontal pole |
| 32/66 | temporalpole | Temporal pole |
| 33/67 | transversetemporal | Transverse temporal cortex |
| 34/68 | insula | Insula |

The Desikan-Killiany atlas divides the left and right hemispheres of the brain into 34 symmetrical regions. In the table, numbers 1 to 34 represent the left hemisphere regions, and 35 to 68 represent the corresponding right hemisphere regions.

**Table S2.** The regions with significant altered cortical thickness (all p < 0.05, FDR corrected) in acute mTBI patients and 1 month follow-up patients compared with HCs.

| Label | Region | T-value | *P* value | *P* value (FDR) |
| --- | --- | --- | --- | --- |
| **Acute mTBI VS. HCs** | | | | |
| 5 | lh_entorhinal | -3.324 | 0.0012 | 0.0236 |
| 39 | rh_entorhinal | -3.582 | 0.0005 | 0.0173 |
| 41 | rh_inferiorparietal | 3.279 | 0.0014 | 0.0236 |
| 61 | rh_superiorfrontal | -3.751 | 0.0003 | 0.0173 |
| **1 month follow-up mTBI VS. HCs** | | | | |
| 6 | lh_fusiform | -3.651 | 0.0004 | 0.0292 |
| 25 | lh_rostralanteriorcingulate | 3.324 | 0.0013 | 0.0432 |

lh, left hemisphere; rh, right hemisphere. T-values were obtained from cortical thickness data (regressed age, sex, and years of education) using two-sample t-tests. All *p* values were adjusted by FDR correction, and were determined based on 2-sided tests.

**Table S3.** The regions with significant altered cortical thickness (all p < 0.05, ANOVA, FDR corrected) in acute mTBI patients and 1 month follow-up patients in Met and Val sub-groups compared with HCs.

| Label | Region | *P* value (FDR) |
| --- | --- | --- |
| **Acute mTBI (Met subgroup) VS. HCs** | | |
| 5 | lh_entorhinal | 0.014 |
| 39 | rh_entorhinal | 0.003 |
| 41 | rh_inferiorparietal | 0.034 |
| 61 | rh_superiorfrontal | 0.001 |
| **Acute mTBI (Val subgroup) VS. HCs** | | |
| 5 | lh_entorhinal | 0.037 |
| 38 | rh_cuneus | 0.031 |
| 41 | rh_inferiorparietal | 0.011 |
| 44 | rh_lateraloccipital | 0.048 |
| **1 month follow-up mTBI (Met subgroup) VS. HCs** | | |
| 6 | lh_fusiform | 0.028 |
| 12 | lh_lingual | 0.003 |
| 25 | lh_rostralanteriorcingulate | 0.004 |
| 32 | lh_temporalpole | 0.018 |
| 48 | rh_middletemporal | 0.01 |
| 51 | rh_parsopercularis | 0.036 |
| 59 | rh_rostralanteriorcingulate | 0.041 |
| **1 month follow-up mTBI (Val subgroup) VS. HCs** | | |
| 6 | lh_fusiform | 0.002 |

lh, left hemisphere; rh, right hemisphere. T-values were obtained from cortical thickness data (regressed age, sex, and years of education) using two-sample t-tests. All *p* values were adjusted by FDR correction, and were determined based on 2-sided tests.
